# Comparative Profiling of Fat-Soluble Nutrients and Antioxidant Indices in Seeds of Six Maple (*Acer*) Types

**DOI:** 10.3390/foods15081279

**Published:** 2026-04-08

**Authors:** Sunleng Chhoeun, Sunyoung Lim, Jeung-Hee Lee, Jung-Ah Shin

**Affiliations:** 1 Department of Marine Convergence Science, Kangwon National University, 7 Jukheon-gil, Gangneung 25457, Gangwon-do, Republic of Korea; chhuoen.sunleng@kangwon.ac.kr (S.C.); isy7516@kangwon.ac.kr (S.L.); 2 Department of Food Processing and Distribution, Kangwon National University, 7 Jukheon-gil, Gangneung 25457, Gangwon-do, Republic of Korea; 3 Department of Food and Nutrition, Daegu University, Gyeongsan 38453, Gyeongsangbuk-do, Republic of Korea; jeunghlee@daegu.ac.kr

**Keywords:** maple seeds, phospholipids, fatty acids, nervonic acid, β-sitosterol, carotenoids, antioxidant indices

## Abstract

Maple (*Acer* spp.) seeds are potential sources of fat-soluble nutrients and bioactive compounds, yet they remain comparatively understudied. This study compared six market-derived *Acer* seed types by quantifying phospholipids (PLs), fatty acids, carotenoids, and phytosterols, and by evaluating total phenolic content (TPC) and DPPH radical scavenging activity in methanolic extracts. Total phospholipid contents varied markedly among samples (17.94–295.87 mg/100 g), with phosphatidylcholine (PC), phosphatidylethanolamine (PE), and phosphatidylinositol (PI) as the predominant classes. Fatty acid profiles were dominated by oleic acid (C18:1) and linoleic acid (C18:2), and nervonic acid (NA; C24:1) was consistently detected in all samples at 0.17–1.88 g/100 g (4.55–7.89% of total fatty acids). β-Sitosterol ranged from 16.58 ± 1.41 to 37.46 ± 1.62 mg/100 g. Carotenoid composition varied among the tested samples, and Jeju red maple showed the highest provitamin A potential, including the exclusive detection of α-carotene and the highest retinol activity equivalent. Antioxidant indices also differed significantly among samples (TPC: 317.89–897.12 mg GAE/100 g; DPPH: 81.37–93.27%), but TPC was not consistently proportional to DPPH activity, suggesting contributions from non-phenolic constituents. Pearson correlation analysis further showed exploratory co-variation patterns among the measured variables across the tested samples. Overall, the tested market-derived *Acer* seed materials exhibited marked compositional diversity and antioxidant potential, supporting their further evaluation as candidate functional food, nutraceutical, and value-added plant lipid resources.

## 1. Introduction

Maples (*Acer* spp.) are widely distributed across the Northern Hemisphere and comprise a diverse genus with strong representation in East Asia [1]. Because of their distinctive form and seasonal coloration, many *Acer* taxa are extensively cultivated as landscaping trees in cities, campuses, and managed green spaces [2,3]. Maples also support established food applications through sap collection and syrup production; prior studies have described that maple sap contains sugars, organic acids, phenolic compounds, amino acids, and minerals that influence syrup composition and quality [4,5,6]. In managed plantings, *Acer* seeds (samaras) are produced annually and are commonly removed during seasonal maintenance. For *Acer truncatum*, fruit biomass of approximately 30 kg per tree has been reported for 20-year-old trees [7]. Chemical studies indicate that *Acer* seeds contain lipids and other nutrients. Maple seed oils have been reported to contain γ-linolenic acid (GLA) and linoleic acid, which was the major fatty acid [8]. In a fatty acid profile analysis of maple seed oils, GLA contents were reported as ~3.275% in *A. platanoides* and ~2.502% in *A. pseudoplatanus*, and linoleic acid were 75.216% and 47.081%, respectively [8]. Across the genus, phytochemical investigations have reported diverse constituents and biological activities for multiple *Acer* species, including phenolic compounds in leaf extracts [3,9].

Nervonic acid (NA; C24:1, n-9) has been reported as a constituent of maple seed oils. Commercial NA has been reported to be derived mainly from *A. truncatum* seeds, which contain approximately 5–6% NA in seed oil [7]. A screening study that measured oil content and fatty-acid composition in seeds of 46 *Acer* species reported that oil contents were below 20% for most tested taxa, with higher oil contents reported for *A. ceriferum* (30.68%), *A. cordatum* (32.05%), and *A. coriaceifolium* (44.84%) [10]. The same study reported NA detection in all 46 samples, NA contents ≥9% in 11 species, and the highest NA content in *A. palmatum* (13.9%) [10]. These reports indicate interspecific variability in both oil content and NA content across *Acer* seeds. In contrast, fewer studies have reported integrated profiles of additional fat-soluble nutrient classes in maple seeds, including phospholipids, carotenoids, and phytosterols, together with antioxidant-related indices derived from phenolic measurements and radical-scavenging assays. Seeds contain storage lipids, and phospholipids represent a defined lipid class relevant to lipid composition and physicochemical behavior in food systems; carotenoids and phytosterols are measurable fat-soluble components with established nutritional relevance. Therefore, comparative datasets that quantify these fat-soluble nutrient classes together with fatty acids (including NA) and antioxidant-related indices across multiple *Acer* taxa are needed to complement the existing fatty-acid-focused literature.

Accordingly, this study quantified phospholipids, fatty acids (with particular emphasis on nervonic acid, NA), carotenoids, and phytosterols, and evaluated total phenolic content and DPPH radical-scavenging activity in seeds from six *Acer* taxa represented by market-derived samples: red maple (*Acer palmatum* var. *amoenum*), maple (*Acer pseudosieboldianum* (Pax.) Kom.), gorosoe (*Acer mono* Maxim.), three-flowered maple with seed coat (*Acer triflorum* Kom.), three-flowered maple with seed coat removed (dehulled) (*Acer triflorum* Kom.), and Jeju red maple (*Acer palmatum* var. *amoenum* ‘Jeju’). Unlike previous maple (*Acer*) seed surveys focused mainly on total oil yield and fatty acid composition, the present study integrates phospholipid, carotenoid, and phytosterol profiling with antioxidant indices to provide a broader compositional dataset relevant to ingredient screening and variety selection.

## 2. Materials and Methods

### 2.1. Material and Reagents

Solvents, including *n*-hexane, ethyl acetate, chloroform, methanol, diethyl ether, petroleum ether, butylated hydroxytoluene (BHT), pyrogallol, and potassium hydroxide (KOH), were purchased from Daejung Chemicals & Metals Co., Ltd. (Siheung, Republic of Korea). Water, tert-butyl methyl ether, isopropanol, acetic acid (AA), and triethylamine (TEA) were obtained from Fisher Scientific Korea Ltd. (Seoul, Republic of Korea). Sodium chloride (NaCl) and sodium carbonate (Na_2_CO_3_) were purchased from Showa Chemicals Co., Ltd. (Tokyo, Japan). 2,2-Diphenyl-1-picrylhydrazyl (DPPH) and gallic acid were obtained from Sigma Chemical Co. (St. Louis, MO, USA). Phospholipid standards, including phosphatidylethanolamine (PE) and phosphatidylcholine (PC), were purchased from Sigma Chemical Co. (St. Louis, MO, USA), whereas sphingomyelin (SM), phosphatidylinositol (PI), L-α-lysophosphatidylethanolamine (LPE), and L-α-lysophosphatidylcholine (LPC) were obtained from Avanti Polar Lipids, Inc. (Alabaster, AL, USA). Carotenoid standards (α-carotene, β-carotene, and β-cryptoxanthin) were purchased from Wako Chemicals Co. (Tokyo, Japan). The β-sitosterol standard, along with hexamethyldisilazane (HMDS), trimethylchlorosilane (TMCS), and 5α-cholestane as an internal standard, was purchased from Sigma Chemical Co. (St. Louis, MO, USA).

### 2.2. Sample Preparation

Six maple seed types, including red maple (RM), maple (M), gorosoe maple (GM), three-flowered maple with seed coat (intact) (TMW1), three-flowered maple without seed coat (dehulled) (TMW2), and Jeju red maple (JRM), were purchased as commercial products from local markets in Korea. Taxonomic designation and country-of-origin information were recorded as stated on the product labeling, and a summary of the taxonomic assignment, sample code, seed coat status, and origin is provided in Table S1. The scientific names and sample labels used in the main text were checked for consistency with Supplementary Table S1. Maple seed samples used in this study were prepared by separating the seeds from commercially purchased maple fruits and removing the attached samara wings prior to analysis. In the case of three-flowered maple (TMW, *Acer triflorum* Kom.), the seed coat exhibited a relatively hard structure; therefore, the seed coat and inner kernel were manually separated (TMW1 and TMW2), homogenized individually using a blender, and analyzed separately. Accordingly, TMW1 was analyzed with the seed coat intact, whereas TMW2 was analyzed after seed coat removal (dehulled). In contrast, the other maple samples had relatively soft seed coats, and the whole seeds were homogenized without prior separation and used for analysis. All samples were thoroughly homogenized using a blender and stored at −20 °C until analysis of fat-soluble nutrients and antioxidant indices.

### 2.3. Folch Extraction and HPLC Analysis for Phospholipid Content

Phospholipids (PLs) were extracted from the samples using the Folch method [11]. For each of the six maple seed samples, a homogenized sample (1 or 0.5 g) was mixed with distilled water (DW), and vortexed. Subsequently, 24 mL of Folch solution (chloroform:methanol = 2:1, *v*/*v*) was added, and the mixture was vortexed for 5 min, sonicated for 5 min, and then centrifuged at 3000 rpm for 10 min to achieve phase separation. The lower (chloroform) layer was collected and passed through an anhydrous sodium sulfate column (ASSC) to remove residual moisture and impurities. The extract was then re-extracted twice with 16 mL of chloroform and 1 mL of methanol. The combined organic phases were concentrated under a nitrogen (N_2_) stream to completely remove the solvents. The crude lipid content was determined and used for PL analysis. PLs were analyzed using an Agilent 1100 series HPLC system equipped with an evaporative light scattering detector (ELSD; Agilent 1100 series, Santa Clara, CA, USA). The ELSD drift tube temperature was set at 60 °C and the nebulizer gas pressure at 1.60 SLM. Separation was performed on a LiChrospher^®^100 Diol column (250 × 4 mm, 5 μm, Merck KGaA, Darmstadt, Germany). The column oven temperature and ELSD evaporation temperature were both maintained at 40 °C. Nitrogen was used as the nebulizing gas at a pressure of 2.5 bar. The injection volume was 10 µL, and the mobile phase flow rate was 1.0 mL/min. The mobile phases consisted of solvent A (*n*-hexane:isopropanol:AA:TEA = 81.42:17:1.5:0.08, *v*/*v*/*v*/*v*) and solvent B (isopropanol:water:AA:TEA = 84.42:14:1.5:0.08, *v*/*v*/*v*/*v*). Calibration curves were constructed using individual PL standard solutions, and PL contents were calculated and expressed as mg/100 g of dry weight, based on duplicate measurements (*n* = 2) for each sample [12].

### 2.4. Acid Hydrolysis and GC Analysis for Fatty Acid Content

Lipid extraction and fatty acid analysis were performed in accordance with the standard operating procedure of the Ministry of Food and Drug Safety (MFDS) [13], the method of Ahn and Shin [14], and the official methods of AOAC International [15]. Lipid was extracted following acid hydrolysis. Briefly, 1 g of a homogenized sample was mixed with 2 mL of 6% pyrogallol solution in ethanol and 1 mL of triundecanoin (5 mg/mL in iso-octane). For hydrolysis, 10 mL of 8.3 M HCl was added, the mixture was vortexed for 30 s, and incubated in a water bath at 80 °C with shaking at 180 rpm for 1 h. After cooling to room temperature, 15 mL of diethyl ether was added and the mixture was vortexed. After phase separation, the upper organic layer was passed through an ASSC and collected. The aqueous phase was re-extracted twice with 15 mL of petroleum ether. The combined organic extracts were concentrated and dried completely under N_2_. Two milliliters of 0.5 N NaOH in methanol was added to the crude fat obtained by acid hydrolysis. Primary methylation was conducted in a water bath at 85 °C with shaking at 180 rpm for 10 min. After cooling, 3 mL of 14% BF_3_ in methanol was added for secondary methylation under the same conditions for 10 min. Subsequently, 3 mL of iso-octane and 1 mL of saturated NaCl solution were added, the mixture was vortexed, and the phases were allowed to separate. The upper organic layer was passed through an ASSC to obtain fatty acid methyl esters (FAMEs), which were analyzed using a gas chromatograph equipped with a flame ionization detector (GC-FID, Hewlett-Packard 6890 series, Agilent Technologies, Santa Clara, CA, USA). Separation was achieved using an SP™-2560 column (100 m × 0.25 mm, 0.2 μm film thickness, Sigma-Aldrich, St. Louis, MO, USA). Helium was used as the carrier gas at a flow rate of 0.7 mL/min. The injector and detector temperatures were set at 225 °C and 285 °C, respectively. An autosampler was used to inject 1 μL in split mode (200:1). Fatty acids were identified using a Supelco 37 Component FAME Mix (CRM 47885, Supelco, Bellefonte, PA, USA), and quantified as fatty acids (g/100 g seeds) [14,16]. Fatty acid content was determined in duplicate (*n* = 2) for each sample.

### 2.5. Saponification of Fat-Soluble Components (β-Sitosterol and Carotenoids)

The fat-soluble components (β-sitosterol and carotenoids) were analyzed according to the method of Ahn and Shin [14]. A homogenized sample (0.5 g) was transferred to an extraction tube, mixed with 4.5 mL of distilled water to obtain a total weight of 5 g, and vortexed until homogeneous. To prevent oxidation, 10 mL of 6% pyrogallol solution in ethanol was added, and the mixture was vortexed. Nitrogen (N_2_) was purged for 1 min to displace air, followed by sonication for 10 min. For saponification, 8 mL of 60% KOH was added, the mixture was vortexed, and N_2_ was purged again for 1 min. The tube was then sealed and incubated in a water bath at 75 °C with shaking at 100 rpm for 1 h. After saponification, the mixture was cooled to room temperature, and 20 mL of 2% NaCl solution and 10 mL of extraction solvent (*n*-hexane: ethyl acetate = 85:15, *v*/*v*) containing 0.01% BHT were added. The mixture was vortexed for 1 min, and the phases were allowed to separate. The upper organic layer was collected, passed through an ASSC, transferred to a 50 mL volumetric flask, and made up to volume with *n*-hexane:ethyl acetate (85:15, *v*/*v*) containing 0.01% BHT for subsequent analysis.

### 2.6. β-Sitosterol Derivatization and GC Analysis

β-Sitosterol was analyzed with minor modifications to the method described by Ahn and Shin [14]. An aliquot (12 mL) of the saponified extract was transferred to a vial and evaporated to dryness under a stream of N_2_. To remove residual moisture, acetone (3 mL) was added, the mixture was vortexed, and the solvent was evaporated; the residue was then dried again under N_2_. The residue was dissolved in DMF (3 mL), and 1 mL of the solution was transferred to a pre-dried test tube (acetone-rinsed and evaporated under N_2_). For derivatization, HMDS (0.2 mL) and TMCS (0.1 mL) were added, the tube was sealed, vortexed for 30 s, and allowed to react at room temperature for 15 min. Distilled water (10 mL) and 1 mL of internal standard solution (0.1 mg/mL 5α-cholestane in heptane) were added, the mixture was vortexed for 1 min. After centrifugation at 2000 rpm for 2 min, the upper organic phase was collected, passed through an ASSC, and transferred to a GC vial. GC-FID analysis was performed using a YL 6100 series gas chromatograph equipped with an HP-Ultra 2 column (25 m × 0.20 mm i.d., 0.33 μm film thickness; Sigma-Aldrich). Nitrogen was used as the carrier gas at a flow rate of 0.8 mL/min. The injector and detector temperatures were set at 280 °C and 300 °C, respectively. A 2 μL aliquot was injected using an autosampler with a split ratio of 20:1. β-Sitosterol content was expressed as mg/100 g dry weight of seed material [14,17]. All analyses were performed in duplicate (*n* = 2).

### 2.7. HPLC Analysis of Carotenoids

Carotenoids in six maple seed samples were analyzed based on the SOP manual for the Food Nutrition Composition Database Construction Project [13] and the method of Ahn and Shin [14]. An aliquot (10 mL) of the saponified extract was transferred to a vial and evaporated to dryness under a stream of N_2_. The residue was reconstituted in 1 mL of chloroform:tert-butyl methyl ether:methanol (1:3: 1, *v*/*v*/*v*) containing 0.01% BHT and filtered through a 0.5 μm hydrophobic PTFE syringe filter (Advantec, Tokyo, Japan). Carotenoids were analyzed using an HPLC system (Agilent 1200 series, Agilent Technology, Waldbrona, Germany) equipped with a UV–visible multiwavelength detector set at 450 nm and fitted with a YMC C30 column (250 × 4.6 mm, 5 μm; YMC Co., Inc., Kyoto, Japan). The mobile phases consisted of solvent A (methanol:tert-butyl methyl ether:methylene chloride:water = 81:14:4:0.05, *v*/*v*/*v*/*v*) and solvent B (methanol:tert-butyl methyl ether:methylene chloride:water:triethylamine = 7:80:10:3:0.05, *v*/*v*/*v*/*v*), which were applied under gradient elution conditions. The flow rate was 1.0 mL/min, and the injection volume was 20 μL. Carotenoid contents were expressed as μg/100 g dry weight and determined in duplicate (*n* = 2) for each sample. Retinol activity equivalents (RAE) were calculated from β-carotene as follows:

μg RAE (μg RAE/100 g) = (β-carotene, μg/100 g)/12(1)

### 2.8. Total Phenolic Content and DPPH Radical Scavenging Activity

Total phenolic content (TPC) and DPPH radical scavenging activity were determined following the method of Singleton and Rossi [18], with minor modifications. For extraction, 0.5 g of each homogenized seed sample was mixed with 5 mL of 100% methanol in a 25 mL vial and vortexed for 10 min. The mixture was centrifuged at 2500rpm for 3 min, and the supernatant was collected. The extraction was repeated twice, and the combined supernatants were concentrated at 38 °C using a vacuum evaporator. The residue was rinsed with 100% methanol, concentrated under a stream of N_2_, reconstituted in 2 mL of methanol, and vortexed prior to analysis. Because methanol was used as the extraction solvent, the TPC and DPPH assays were interpreted as antioxidant indices of methanolic extracts rather than direct measurements of the lipid fraction. However, because several fat-soluble constituents were extracted using methanol-containing solvent systems, including the Folch solvent system (chloroform:methanol, 2:1, *v*/*v*), these antioxidant indices may still reflect, at least in part, compositional variation associated with lipid-soluble bioactives.

TPC was determined using the Folin–Ciocalteu method, following the official methods of AOAC International [19] with minor modifications. An aliquot of methanolic extract (0.2 mL) was mixed with 1 mL of 10% Folin–Ciocalteu reagent and 4 mL of distilled water, vortexed, and then 0.8 mL of 7.5% Na_2_CO_3_ solution was added. After incubation in the dark for 30 min, absorbance was measured at 715 nm. A gallic acid calibration curve (0.0078–1.0 mg/mL) was prepared and used for quantification. Results were expressed as mg gallic acid equivalents per 100 g dry weight (mg GAE/100 g), and all measurements were performed in triplicate (*n* = 3).

DPPH radical scavenging activity was determined according to the method of Blois [20]. The DPPH solution (0.15 mM) was prepared by dissolving 0.0059 g of DPPH in 100 mL of ethanol. Ascorbic acid (0.1 and 1 mg/mL) and BHT (0.1 and 1 mg/mL) were used as positive controls and prepared in 100% methanol. For the assay, 0.5 mL of each methanolic extract was mixed with 4 mL of 0.15 mM DPPH solution and incubated in the dark for 30 min. Absorbance was measured at 517 nm, and radical scavenging activity was calculated as follows:

DPPH radical scavenging activity (%) = (1 − S/C) × 100 (2)

where S is the absorbance of the sample and C is the absorbance of the control at 517 nm.

### 2.9. Statistical Analysis

Data are presented as mean ± standard deviation (SD) of replicate measurements. Differences among samples were evaluated by one-way analysis of variance (ANOVA), followed by Tukey’s honestly significant difference (HSD) post hoc test. Correlation analysis based on the six maple seed samples was treated as exploratory because of the limited sample size. Differences were considered statistically significant at *p* < 0.05. All statistical analyses were performed using SPSS Statistics (version 29.0; IBM Corp., Armonk, NY, USA).

## 3. Results and Discussion

### 3.1. HPLC Analysis of Phospholipid Contents

Lipid and phospholipid (PL) contents of the six maple seed samples are shown in Table 1. Total lipid content obtained by the Folch extraction method ranged from 4.82 ± 0.18% to 32.70 ± 0.32%, with significant differences among samples (*p* < 0.05). Likewise, total PL content differed significantly, ranging from 17.94 ± 1.89 to 295.87 ± 3.01 mg/100 g (*p* < 0.05). These results indicate marked sample-dependent variation in membrane lipid composition and among the tested market-derived maple seed materials.

GM showed the highest total phospholipid (PL) content (295.87 ± 3.01 mg/100 g), which was approximately 16-fold higher than that of RM (17.94 ± 1.89 mg/100 g) and M (21.55 ± 1.32 mg/100 g). GM also exhibited the highest lipid content (32.70 ± 0.32%), indicating marked compositional differences among the tested market-derived materials. The elevated PL content in GM may be associated with membrane lipid remodeling related to seed physiological status [21]. In contrast, RM and M consisted entirely of PI within the detected PL fraction, indicating a predominance of PI in these samples. Given the role of PI in cellular signaling and membrane trafficking, this pattern may reflect sample-specific differences in lipid organization [22].

A putative SM-like peak (putative SM) was detected only in TMW2 and JRM, accounting for 9.55% and 5.61% of the individual PL fraction, respectively. The absence of detectable putative SM in TMW1 but its presence in TMW2 may be related to seed coat removal, because the relatively thick seed coat of three-flowered maple may reduce oil extraction efficiency. Thus, removal of the seed coat in TMW2 may have increased lipid recovery and facilitated detection of the putative SM-like peak, although this interpretation remains speculative and requires further analytical confirmation. Although SM is generally considered rare in plants, recent lipidomics studies have reported sphingomyelin-related lipid signals in seed tissues. For example, a spatial lipidomics study of germinating mung bean seeds identified sphingomyelin-related lipid signals in plumule tissue, suggesting that an SM-like signal or closely related sphingolipids may occur in specific seed tissues during germination [23]. In the present study, representative chromatograms and orthogonal confirmation data were not available; therefore, the putative SM assignment should be interpreted cautiously. Accordingly, the observed putative SM signal in TMW2 and JRM may reflect sample-dependent differences in sphingolipid-related components. In contrast, no corresponding signal was detected in RM, M, GM, or TMW1. Representative chromatograms and peak-identification materials supporting the present phospholipid assignment are provided in Supplementary Figures S1 and S2.

Among the samples in which multiple PL classes were detected, PC was the predominant PL, followed by PE and PI. The contents of PC, PE, and PI ranged from 0 to 143.82 ± 2.97 mg/100 g, 0 to 46.26 ± 5.48 mg/100 g, and 13.55 ± 0.98 to 105.79 ± 0.50 mg/100 g, respectively, and differed significantly among samples (*p* < 0.05). Aremu et al. [24] reported PC, PE, and PI contents of 195.03, 21.12, and 59.87 mg/100 g, respectively, in breadfruit seeds, which are broadly comparable to the ranges observed in the present study. Variations in PL composition among seeds have been attributed to differences in analytical methods, genotype, and cultivation environment [25,26].

### 3.2. GC Analysis of Fatty Acid Contents

**Figure 1:**
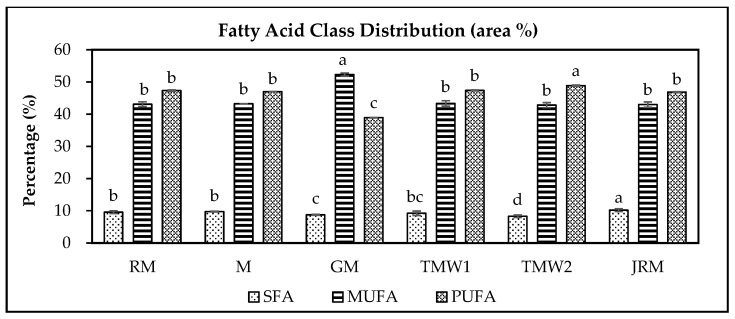
Fatty acid class distribution in six maple seed samples. Values are expressed as mean ± standard deviation (*n* = 2); ^a–d^ Different letters indicate significant differences among samples within the same fatty acid class (Tukey’s HSD test, *p* < 0.05); SFA: saturated fatty acids (palmitic, stearic, and behenic acids); MUFA: monounsaturated fatty acids (gondoic, erucic, oleic, and nervonic acids); PUFA: polyunsaturated fatty acids (linoleic, γ-linolenic acid, and α-linolenic acids).

As shown in Table 2, total fat content determined by acid hydrolysis ranged from 3.64 ± 0.12% to 29.87 ± 0.30% and differed significantly among the maple seed samples (*p* < 0.05). GM and TMW2 contained more than 20% total fat, indicating relatively high lipid accumulation in these taxa. Total fatty acid content also varied significantly, ranging from 3.16 ± 0.13 to 30.77 ± 1.26 g/100 g (*p* < 0.05). Consistent with these differences, the fatty acid profiles of the six *Acer* seed samples (Figure 1) showed substantial sample-dependent variation in the distribution of saturated fatty acids (SFAs), monounsaturated fatty acids (MUFAs), and polyunsaturated fatty acids (PUFAs).

MUFAs were the predominant fatty acid class in all samples, ranging from approximately 42.69% to 52.17% (*p* < 0.05), with GM showing the highest proportion. This pattern is consistent with previous reports for woody plant seeds, in which oleic acid (C18:1) is typically the major unsaturated fatty acid [27]. PUFAs were the second most abundant class, ranging from approximately 38.59% to 48.39% (*p* < 0.05), whereas SFAs accounted for a relatively small proportion of total fatty acids, approximately 8–10%, indicating more limited variation among samples than the unsaturated fatty acid classes. The major SFAs were palmitic acid (C16:0) and stearic acid (C18:0), both of which are important structural components of plant membranes and storage lipids [28]. Notably, nervonic acid (NA; C24:1), a distinctive very-long-chain monounsaturated fatty acid, was detected in all maple seed samples (Table 2) at levels ranging from 0.17 ± 0.01 to 1.88 ± 0.12 g/100 g, corresponding to 4.55–7.89% of total fatty acids. NA has been reported to be an important component of myelin lipids and to be required for myelin maintenance, particularly during early development [29]. The consistent presence of NA in all samples suggests that maple seeds may represent a potential plant source of this nutritionally relevant fatty acid. High MUFA levels may also contribute to membrane fluidity and the nutritional quality of plant-derived lipids [30]. Linoleic acid (C18:2) was the dominant PUFA in all samples, whereas α-linolenic acid (C18:3) was present at comparatively lower levels. Because PUFAs play important roles in plant stress responses, membrane function, and signaling pathways [31], the relatively high PUFA proportions observed in several maple seed samples may indicate enhanced membrane flexibility and adaptive capacity under low-temperature or oxidative stress conditions [32].

### 3.3. GC Analysis of β-Sitosterol Contents

**Figure 2:**
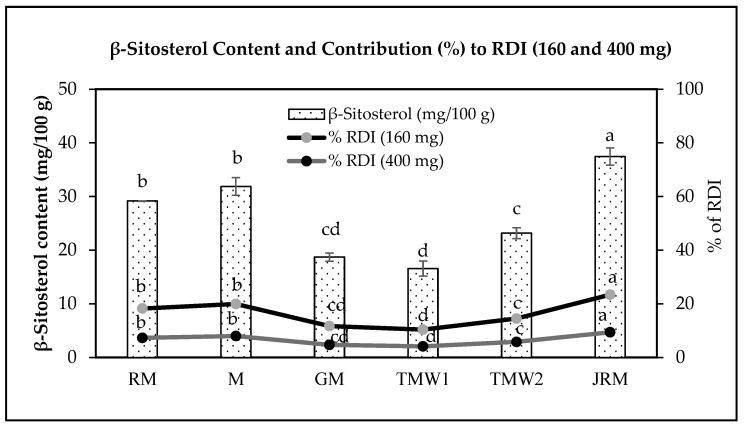
β-Sitosterol content (mg/100 g) and estimated contribution (%) to the recommended daily intake (RDI; 160 mg and 400 mg) in six maple seed samples. Values are expressed as mean ± standard deviation (*n* = 2); ^a–d^ Different letters indicate significant differences in β-sitosterol content among samples (Tukey’s HSD test, *p* < 0.05); RDI: recommended dietary intake.

The β-sitosterol contents of the six maple seed samples are presented in Figure 2. β-Sitosterol levels differed significantly among samples, ranging from 16.58 ± 1.41 to 37.46 ± 1.62 mg/100 g dry weight (*p* < 0.05). JRM showed the highest β-sitosterol content, whereas TMW1 showed the lowest. The estimated contribution of β-sitosterol to the recommended daily intake (RDI) also varied among samples, corresponding to 10.36–23.41% for the 160 mg criterion and 4.15–9.37% for the 400 mg criterion. 

β-Sitosterol is a phytosterol widely distributed in plant-derived foods, including rice bran, wheat germ, peanuts, corn oil, and soybeans. In soybeans, total phytosterol content has been reported to range from 19.25 to 35.34 mg/100 g [33], which is comparable to the β-sitosterol levels observed in the present study. Because β-sitosterol has been associated with cholesterol-lowering effects and other bioactivities, including anti-inflammatory, antibacterial, antifungal, and antioxidant properties [34], the relatively high levels detected in maple seeds may indicate their nutritional and functional potential. In particular, the high β-sitosterol content of JRM suggests that this seed type may be a promising source of phytosterols among the *Acer* samples examined.

### 3.4. HPLC Analysis of Carotenoid Contents

Carotenoid contents of the six maple seed samples are presented in Table 3. Lutein was the predominant carotenoid and was detected in all samples, ranging from 53.21 ± 3.63 to 498.22 ± 4.34 μg/100 g. In contrast, β-carotene ranged from 2.48 ± 0.15 to 35.25 ± 0.24 μg/100 g and differed significantly among samples (*p* < 0.05). Zeaxanthin and β-cryptoxanthin were detected only in GM, TMW2, and JRM, whereas α-carotene was detected exclusively in JRM (3.72 ± 0.28 μg/100 g). Because retinol was not determined in the present study, retinol activity equivalents (RAE) were calculated from β-carotene only. Under this approach, JRM showed the highest RAE value (3.47 μg RAE/100 g), whereas all other samples remained below 2.0 μg RAE/100 g.

These results indicate marked sample-dependent variation in carotenoid composition among the tested market-derived *Acer* seed samples. In particular, JRM exhibited the highest overall carotenoid accumulation, including the highest lutein and β-carotene contents, and was the only sample in which α-carotene was detected. This pattern is consistent with its highest RAE value and suggests relatively greater provitamin A potential than that of the other tested samples [35]. Although the RAE values were low compared with those of vitamin A-rich foods, the detection of provitamin A carotenoids in seeds is noteworthy because seeds are not generally considered major storage organs for carotenoids [36]. The distinct carotenoid profiles observed among the tested samples may therefore reflect sample-dependent differences in carotenoid biosynthesis, accumulation, and deposition in reproductive tissues.

### 3.5. Comparison of TPC and DPPH Antioxidant Activity

As shown in Table 4, total phenolic content (TPC) differed significantly among the maple seed samples, ranging from 317.89 ± 28.58 to 897.12 ± 35.88 mg GAE/100 g (*p* < 0.05). GM exhibited the highest TPC, whereas TMW2 showed the lowest. DPPH radical scavenging activity also varied significantly among samples, ranging from 81.37 ± 3.54% to 93.27 ± 0.56% (*p* < 0.05). JRM showed the highest DPPH activity, followed closely by RM, whereas GM showed the lowest activity. When expressed relative to the DPPH scavenging activity of 0.1 mg/mL ascorbic acid, the maple seed extracts showed high relative activities ranging from 84.43% to 96.78%**,** indicating substantial radical scavenging capacity under the present assay conditions.

The TPC values of maple seeds in the present study were higher than those reported for moringa and pumpkin seeds (212.8 ± 10.05 and 152.00 ± 53.00 mg GAE/100 g, respectively) [37,38], suggesting that *Acer* seeds may represent a relatively rich source of phenolic compounds. However, TPC did not consistently correspond to DPPH radical scavenging activity. For example, JRM showed relatively low TPC (333.51 mg GAE/100 g) but the highest DPPH activity (93.27%), whereas GM showed the highest TPC (897.12 mg GAE/100 g) but comparatively low DPPH activity (83.59%). This discrepancy suggests that TPC alone does not fully explain antioxidant performance in these samples [39]. Because the DPPH assay reflects overall radical scavenging capacity rather than phenolic content alone [40], the contribution of non-phenolic antioxidants should also be considered. In contrast, the Folin–Ciocalteu assay primarily measures electron-transfer-based reducing capacity, and although it responds strongly to many phenolic compounds, several non-phenolic antioxidants show limited reactivity in this assay [39]. Accordingly, the high DPPH activity observed in JRM and RM may reflect the presence of additional antioxidant constituents beyond phenolics. The reference compounds further contextualize the antioxidant potency of the maple seed extracts. Ascorbic acid (0.1–1.0 mg/mL) showed consistently high DPPH scavenging activity (96.05–96.37%), whereas BHT exhibited concentration-dependent activity, with 1.0 mg/mL BHT showing 83.79 ± 0.06% scavenging activity and 0.1 mg/mL BHT showing only 23.52 ± 1.67%**.** These results confirm that the antioxidant activity of the maple seed extracts was comparable to that of 0.1 mg/mL ascorbic acid and 1.0 mg/mL BHT under the present assay conditions. Collectively, these findings indicate that *Acer* seeds possess substantial radical scavenging activity and support their potential as functional food resources. Because antioxidant activity was evaluated only by TPC and DPPH using methanolic extracts, the present data should be interpreted as comparative antioxidant indices rather than as a complete characterization of antioxidant capacity. Additional assays such as ABTS, FRAP, or CUPRAC would be valuable in future studies.

### 3.6. Correlation Among Fat-Soluble Nutrients, Phytochemicals, and Antioxidant Activity

Pearson correlation analysis revealed several exploratory relationships among fat-soluble nutrients, phytochemicals, and antioxidant activity in the tested maple seed samples, as summarized in Table 5. MUFA and PUFA showed a strong inverse association (r = −0.984, *p* < 0.05), which may reflect compositional opposition between these two major unsaturated fatty acid classes within the present dataset. Total phospholipids (PLs) also tended to be positively associated with MUFA (r = 0.781) and negatively associated with PUFA (r = −0.789).

Phenolic compounds showed a significant negative correlation with PUFA (r = −0.820, *p* < 0.05). This pattern may be relevant because PUFA-rich matrices are generally more susceptible to oxidative deterioration [41]. Although interactions between polyphenols and other dietary compounds can influence antioxidant behavior [42], the phenolic compound–DPPH relationship in this study was weakly negative (r = −0.184), suggesting that antioxidant responses in these samples may not be explained by phenolic content alone. In addition, β-sitosterol showed a positive correlation with DPPH (r = 0.807), which is in line with previous reports describing concentration-dependent DPPH inhibition by β-sitosterol [43].

Overall, these findings should be interpreted as exploratory compositional relationships in a limited set of market-derived samples rather than as mechanistic evidence. Nevertheless, they may be useful for generating hypotheses and guiding future targeted studies on compositional variation and functional potential.

## 4. Conclusions

In this study, fat-soluble components and antioxidant activities were comprehensively compared among six market-derived maple (*Acer*) seed types by quantifying PLs, fatty acids, carotenoids, phytosterols, TPC, and DPPH radical scavenging activity. Extending previous studies that focused mainly on fatty acid composition in selected *Acer* species, the present work provides a more integrated compositional dataset covering major lipid classes, lipophilic micronutrients, and antioxidant indices. Total phospholipid contents varied markedly among samples (17.94–295.87 mg/100 g, *p* < 0.05), with PC, PE, and PI as the predominant PL classes. Fatty acid profiles were dominated by oleic acid (C18:1) and linoleic acid (C18:2), and nervonic acid (C24:1) was consistently detected in all samples. Among the tested materials, Jeju red maple showed the highest β-sitosterol content and the greatest provitamin A potential, including the exclusive detection of α-carotene and the highest retinol activity equivalent. In contrast, gorosoe maple showed relatively high levels of major fatty acids, including oleic, linoleic, and nervonic acids, whereas three-flowered maple without seed coat exhibited a distinct profile with relatively elevated γ-linolenic acid, suggesting potential value for specialized nutraceutical or cosmetic applications within the tested materials. These findings indicate clear sample-dependent differences among the market-derived maple seed materials analyzed here and suggest that such materials may be selected according to targeted compositional traits, while broader biological generalization at the species level should be made cautiously. Antioxidant indices also differed significantly among samples, with TPC ranging from 317.89 to 897.12 mg GAE/100 g and DPPH radical scavenging activity ranging from 81.37% to 93.27% (*p* < 0.05). Notably, the lack of a consistent proportional relationship between TPC and DPPH activity suggests that antioxidant capacity in these tested materials is influenced by both phenolic and non-phenolic constituents. This interpretation was further supported by correlation analysis, which revealed exploratory co-variation patterns among lipid classes, phytochemicals, and antioxidant activity within the tested samples.

Overall, the present study demonstrates pronounced sample-dependent diversity in the lipid composition, phytochemical profile, and antioxidant potential of the tested market-derived *Acer* seed materials. These findings provide a useful reference framework for future studies and support further evaluation of these materials as candidate functional food, nutraceutical, or value-added plant lipid resources. From an application perspective, however, the present findings should be interpreted as a compositional basis for future ingredient development rather than as direct evidence of immediate industrial feasibility or as firm species-wide conclusions. In particular, the extraction and analytical workflows used in this study, including chloroform:methanol, hexane, diethyl ether, petroleum ether, and derivatization-based GC procedures, were intended for compositional analysis and are not directly transferable to food-grade industrial processing. Future development would therefore require evaluation of food-compatible extraction approaches, solvent recovery, storage stability, and practical handling of maple samaras and seeds, including collection, dehulling, and storage.

## Figures and Tables

**Table 1:** Phospholipid composition of seeds from six maple (*Acer*) types.

Samples	Lipid Contents (%) ^2^	Phospholipid (mg/100 g Dry Weight; % of Individual PLs)				
		PE	PC	Putative SM*	PI	Total PLs
RM	13.46 ± 0.17 ^1,c^	ND ^3^	ND	ND	17.94 ± 1.89 ^d^ (100%)	17.94 ± 1.89 ^d^
M	14.37 ± 0.76 ^c^	ND	ND	ND	21.55 ± 1.32 ^d^ (100%)	21.55 ± 1.32 ^d^
GM	32.70 ± 0.32 ^a^	46.26 ± 5.48 ^a^ (15.64%)	143.82 ± 2.97 ^a^ (48.61%)	ND	105.79 ± 0.50 ^a^ (35.76%)	295.87 ± 3.01 ^a^
TMW1	4.82 ± 0.18 ^d^	6.04 ± 0.28 ^cd^ (18.67%)	12.76 ± 0.09 ^d^ (39.43%)	ND	13.55 ± 0.98 ^d^ (41.87%)	32.36 ± 0.60 ^d^
TMW2	27.66 ± 2.12 ^b^	13.28 ± 0.44 ^c^ (14.23%)	30.54 ± 2.13 ^c^ (32.73%)	8.91 ± 0.25 ^b^ (9.55%)	40.59 ± 1.62 ^c^ (43.50%)	93.32 ± 3.05 ^c^
JRM	11.75 ± 0.61 ^c^	23.31 ± 1.7 ^b^ (11.73%)	75.41 ± 4.92 ^b^ (37.95%)	11.15 ± 0.71 ^a^ (5.61%)	88.85 ± 5.61 ^b^ (44.71%)	198.71 ± 12.94 ^b^

^1^ Data are presented as mean ± standard deviation (SD) (*n* = 2); ^2^ Lipid content was determined using the Folch extraction method; ^3^ ND: not detected; ^a–d^ Means within the same column followed by different lowercase letters are significantly different among the six maple seed samples (Tukey’s HSD test, *p* < 0.05). Abbreviations: PE, phosphatidylethanolamine; PC, phosphatidylcholine; Putative SM*, putative sphingomyelin-like peak (tentative assignment based on retention time matching only and not structurally confirmed); PI, phosphatidylinositol; Total PLs, total phospholipids.

**Table 2:** Fatty acid (FA) contents of seeds from six maple (*Acer*) types.

Fatty Acid Content (FA g/100 g Seeds)							
Fatty Acids		Maple Seed Samples					
		RM	M	GM	TMW1	TMW2	JRM
Saturated fatty acids (SFAs)	C14:0	ND ^2^	0.01 ± 0 ^a^	0.01 ± 0 ^a^	ND	ND	0.01 ± 0 ^a^
	C16:0	0.55 ± 0.07 ^1,c^	0.57 ± 0.02 ^c^	1.46 ± 0.04 ^a^	0.16 ± 0.01 ^d^	0.97 ± 0.11 ^b^	0.47 ± 0.01 ^c^
	C17:0	0.01 ± 0 ^bc^	0.01 ± 0 ^bc^	0.02 ± 0 ^ab^	0 ± 0 ^c^	0.03 ± 0 ^a^	0.01 ± 0 ^bc^
	C18:0	0.22 ± 0.03 ^c^	0.25 ± 0.01 ^c^	0.75 ± 0.03 ^a^	0.07 ± 0 ^d^	0.44 ± 0.05 ^b^	0.22 ± 0 ^c^
	C20:0	0.03 ± 0 ^dc^	0.03 ± 0 ^bc^	0.07 ± 0 ^a^	0.01 ± 0 ^d^	0.04 ± 0.01 ^b^	0.03 ± 0 ^bc^
	C22:0	0.12 ± 0.02 ^b^	0.13 ± 0 ^b^	0.24 ± 0.01 ^a^	0.02 ± 0 ^c^	0.12 ± 0.02 ^b^	0.12 ± 0.01 ^b^
	C23:0	0.01 ± 0 ^a^	0.01 ± 0 ^a^	0.01 ± 0 ^a^	ND	0.01 ± 0 ^a^	0.01 ± 0 ^a^
	C24:0	0.06 ± 0.01 ^b^	0.07 ± 0 ^b^	0.1 ± 0 ^a^	0.01 ± 0 ^c^	0.05 ± 0.01 ^b^	0.06 ± 0 ^b^
Monounsaturated fatty acids (MUFAs)	C16:1	ND	ND	0.03 ± 0 ^a^	ND	0.01 ± 0 ^b^	0.01 ± 0 ^b^
	C17:1	ND	ND	0.01 ± 0 ^a^	ND	0.02 ± 0 ^a^	ND
	C18:1 (n-9)	1.4 ± 0.2 ^c^	1.48 ± 0.04 ^c^	6.79 ± 0.24 ^a^	0.58 ± 0.02 ^c^	3.9 ± 0.5 ^b^	1.31 ± 0.01 ^c^
	C20:1	0.51 ± 0.08 ^c^	0.59 ± 0.02 ^c^	2.37 ± 0.11 ^a^	0.21 ± 0.01 ^c^	1.31 ± 0.19 ^b^	0.44 ± 0.01 ^c^
	C22:1 (n-9)	1.84 ± 0.31 ^bc^	1.93 ± 0.05 ^bc^	5.12 ± 0.28 ^a^	0.43 ± 0.03 ^d^	2.58 ± 0.42 ^b^	1.52 ± 0.07 ^c^
	C24:1 (n-9)	0.88 ± 0.16 ^b^	0.89 ± 0.02 ^b^	1.88 ± 0.12 ^a^	0.17 ± 0.01 ^c^	0.98 ± 0.17 ^b^	0.68 ± 0.04 ^b^
Polyunsaturated fatty acids (PUFAs)	C18:2 (n-6)	4.38 ± 0.61 ^c^	4.65 ± 0.11 ^c^	10.89 ± 0.41 ^a^	1.38 ± 0.04 ^d^	9.15 ± 1.16 ^b^	3.75 ± 0.05 ^c^
	C18:3 (n-6)	0.17 ± 0.02 ^bc^	0.15 ± 0 ^bc^	0.24 ± 0 ^b^	0.08 ± 0 ^c^	0.54 ± 0.06 ^a^	0.14 ± 0 ^c^
	C18:3 (n-3)	0.39 ± 0.06 ^b^	0.37 ± 0.01 ^b^	0.65 ± 0.02 ^a^	0.01 ± 0 ^c^	0.08 ± 0.01 ^c^	0.31 ± 0 ^b^
	C20:2	0.04 ± 0.01 ^bc^	0.05 ± 0 ^b^	0.09 ± 0 ^a^	0.01 ± 0 ^d^	0.08 ± 0.01 ^a^	0.03 ± 0 ^c^
	C22:2 (n-6)	0.01 ± 0 ^a^	0.02 ± 0 ^a^	0.02 ± 0 ^a^	0 ± 0 ^b^	0.02 ± 0 ^a^	0.01 ± 0 ^ab^
Total fatty acid contents		10.64 ± 1.59 ^c^	11.23 ± 0.29 ^c^	30.77 ± 1.26 ^a^	3.16 ± 0.13 ^d^	20.33 ± 2.73 ^b^	9.11 ± 0.21 ^c^
Fat contents (%) ^3^		12.27 ± 0.61 ^cd^	12.84 ± 0.72 ^c^	29.87 ± 0.3 ^a^	3.64 ± 0.12 ^e^	27.9 ± 0.22 ^b^	11.06 ± 0.06 ^d^

^1^ Data are presented as mean ± standard deviation (SD) (*n* = 2); ^2^ ND: not detected; ^3^ Fat content was determined using the acid hydrolysis method; ^a–e^ Means within the same row followed by different lowercase letters are significantly different among the six maple seed samples (Tukey’s HSD test, *p* < 0.05).

**Table 3:** Carotenoid contents and retinol activity equivalents (RAE) in seeds of six maple (*Acer*) types.

Samples	Lutein	Zeaxanthin	β-Cryptoxanthin (µg/100 g)	α-Carotene (µg/100 g)	β-Carotene (µg/100 g)	RAE (µg RAE/100 g)
RM	79.94 ± 1.03 ^1,d^	ND ^2^	ND	ND	3.50 ± 0.25 ^c^	0.29 ^c^
M	53.21 ± 3.63 ^e^	ND	ND	ND	2.48 ± 0.15 ^c^	0.21 ^c^
GM	217.33 ± 7.93 ^b^	2.14 ± 0.10 ^b^	1.60 ± 0.12 ^b^	ND	20.75 ± 1.25 ^b^	1.86 ^b^
TMW1	104.32 ± 7.90 ^c^	ND	ND	ND	3.62 ± 0.05 ^c^	0.30 ^c^
TMW2	225.38 ± 2.61 ^b^	2.15 ± 0.01 ^b^	2.67 ± 0.11 ^a^	ND	18.35 ± 0.97 ^b^	1.75 ^b^
JRM	498.22 ± 4.34 ^a^	4.23 ± 0.51 ^a^	2.65 ± 0.16 ^a^	3.72 ± 0.28 ^a^	35.25 ± 0.24 ^a^	3.47 ^a^

^1^ Data are presented as mean ± standard deviation (*n* = 2); ^2^ ND: not detected; ^a–e^ Means within the same column followed by different lowercase letters are significantly different among the six maple seed samples (Tukey’s HSD test, *p* < 0.05); RAE: retinol activity equivalents.

**Table 4:** Total phenolic content (TPC) and DPPH radical scavenging activity in seeds of six maple (*Acer*) types.

Samples	TPC (mg GAE/100 g)	DPPH Radical Scavenging Activity (%)	Relative Activity (%) ^2^
RM	506.42 ± 30.99 ^1,bc^	92.69 ± 0.26 ^a^	96.18 ^a,^
M	695.12 ± 159.26 ^b^	90.09 ± 0.57 ^a^	93.4 8 ^a^
GM	897.12 ± 35.88 ^a^	83.59 ± 0.13 ^bc^	86.74 ^bc^
TMW1	538.27 ± 14.26 ^b^	85.56 ± 0.65 ^b^	88.78 ^b^
TMW2	317.89 ± 28.58 ^c^	81.37 ± 3.54 ^c^	84.43 ^c^
JRM	333.51 ± 18.98 ^c^	93.27 ± 0.56 ^a^	96.78 ^a^
Ascorbic acid 1 mg/mL	-	96.37 ± 0.00	-
Ascorbic acid 0.1 mg/mL	-	96.05 ± 0.06	-
BHT 1 mg/mL	-	83.79 ± 0.06	-
BHT 0.1 mg/mL	-	23.52 ± 1.67	-

^1^ Data are presented as mean ± standard deviation (*n* = 3); ^2^ Relative activity (%) was calculated as: (DPPH activity of sample/DPPH activity of ascorbic acid at 0.1 mg/mL) × 100; ^a–c^ Means within the same column followed by different lowercase letters are significantly different among the six maple seed samples (Tukey’s HSD test, *p* < 0.05); GAE: gallic acid equivalents; BHT: butylated hydroxytoluene.

**Table 5:** Pearson correlations among fat-soluble nutrients and phytochemicals in six maple (*Acer*) seeds types.

Pearson Correlation Matrix Among Measured Components									
	Total PLs	SFA	MUFA	PUFA	Carotene	Xanthophyll	β-Sitosterol	Phenolic Compounds	DPPH
**Total PLs**	1								
**SFA**	−0.172	1							
**MUFA**	0.781	−0.367	1						
**PUFA**	−0.789	0.197	−0.984 **	1					
**Carotene**	0.752	0.092	0.176	−0.201	1				
**Xanthophyll**	−0.803	0.297	−0.474	0.440	−0.788	1			
**β-Sitosterol**	−0.080	0.754	−0.464	0.349	0.368	−0.190	1		
**Phenolic compounds**	0.327	−0.116	0.797	−0.820 *	−0.336	−0.003	−0.396	1	
**DPPH**	−0.236	0.943 **	−0.401	0.245	0.043	0.255	0.807	−0.184	1

** Correlation is significant at *p* < 0.01; * Correlation is significant at *p* < 0.05; r: correlation coefficient; Total PLs: total phospholipids; SFA: saturated fatty acids; MUFA: monounsaturated fatty acids; PUFA: polyunsaturated fatty acids; DPPH: 2,2-diphenyl-1-picrylhydrazyl radical scavenging activity.

## Data Availability

The original contributions presented in the study are included in the article. Further inquiries can be directed to the corresponding author.

## Supplementary Materials

The following supporting information can be downloaded at: https://www.mdpi.com/article/10.3390/foods15081279/s1, Figure S1: Representative HPLC-ELSD chromatogram of phospholipid classes; Figure S2: Representative HPLC-ELSD chromatograms of phospholipid classes in TMW1 (*Acer triflorum* Kom.; blue trace, upper chromatogram) and TMW2 (*Acer triflorum* Kom.; red trace, lower chromatogram). Table S1: Taxonomic assignment (as labeled) and seed coat status of the six market-derived *Acer* seed samples.

## References

[1] van Gelderen D.M., de Jong P.C., Oterdoom H.J., Dudley T.R. (1994). Maples of the World.

[2] Wang D. (2021). Seasonal color matching method of ornamental plants in urban landscape construction. Open Geosci..

[3] Wu B., Gao Y., Shen J., He C., Liu H., Peng Y., Zhang C., Xiao P. (2016). Traditional uses, phytochemistry, and pharmacology of the genus *Acer* (maple): A review. J. Ethnopharmacol..

[4] Perkins T.D., Van den Berg A. (2009). Maple syrup—Production, composition, chemistry, and sensory characteristics. Adv. Food Nutr. Res..

[5] Pollard J.K., Sproston T. (1954). Nitrogenous constituents of sap from *Acer saccharum*. Plant Physiol..

[6] Lagacé L., Gagnon D., Bureau S. (2015). Biochemical composition of maple sap among constituents. J. Food Compos. Anal..

[7] Qiao Q., Wang X., Ren H., An K., Feng Z., Cheng T., Sun Z. (2019). Oil content and nervonic acid content of *Acer truncatum* seeds from 14 regions in China. Hortic. Plant J..

[8] Hovanet M.V., Dociu N., Dinu M., Ancuceanu R., Morosan E., Oprea E. (2015). A comparative physico-chemical analysis of *Acer platanoides* and *Acer pseudoplatanus* seed oils. Rev. Chim..

[9] Kausar F., Farooqi M.A., Farooqi H.M.U., Salih A.R.C., Khalil A.A.K., Kang C.W., Mahmoud M.H., Batiha G.E.-S., Choi K.H., Mumtaz A.S. (2021). Phytochemical investigation, antimicrobial, antioxidant and anticancer activities of *Acer cappadocicum* gled. Life.

[10] He X., Li D.Z., Tian B. (2021). Diversity in seed oil content and fatty acid composition in *Acer* species with potential as sources of nervonic acid. Plant Divers..

[11] Folch J., Lees M., Stanley G.H.S. (1957). A simple method for the isolation and purification of total lipides from animal tissues. J. Biol. Chem..

[12] Ministry of Food and Drug Safety (MFDS) (2025). General Analytical Method: Codes 8.6.3.2.3.1. Phospholipid.

[13] Ministry of Food and Drug Safety (MFDS) (2025). Korea Food Code: Acid Hydrolysis (2.1.5.1.2).

[14] Ahn T.Y., Shin J. (2023). Analysis of fat-soluble nutrients (tocopherol, retinol, β-carotene, cholesterol, and fatty acids) in seafood restaurant foods commonly consumed in Korea. J. Korean Soc. Food Sci. Nutr..

[15] AOAC International (2001). Official Methods of Analysis of AOAC International.

[16] Ministry of Food and Drug Safety (MFDS) (2025). Korea Food Code: Fatty Acids (2.1.5.4).

[17] Phillips K.M., Ruggio D.M., Bailey J.A. (1999). Precise quantitative determination of phytosterols, stanols, and cholesterol metabolites in human serum by capillary gas-liquid chromatography. J. Chromatogr. B.

[18] Singleton V.L., Rossi J.A. (1956). Colorimetry of total phenolics with phosphomolybdic-phosphotungstic acid reagent. Am. J. Enol. Vitic..

[19] AOAC International (2017). Official Methods of Analysis of AOAC International.

[20] Blois M.S. (1958). Antioxidant determinations by the use of a stable free radical. Nature.

[21] Yu X., Li A., Li W. (2015). How membranes organize during seed germination: Three patterns of dynamic lipid remodeling define chilling resistance and affect plastid biogenesis. Plant Cell Environ..

[22] Okazaki Y., Otsuki H., Narisawa T., Kobayashi M., Sawai S., Kamide Y., Kusano M., Aoki T., Yokota Hirai M., Saito K. (2013). A new class of plant lipid is essential for protection against phosphorus depletion. Nat. Commun..

[23] Xie P., Chen J., Wu P., Cai Z. (2023). Spatial lipidomics reveals lipid changes in the cotyledon and plumule of mung bean seeds during germination. J. Agric. Food Chem..

[24] Aremu M.O., Haruna A., Oko O.J., Ortutu S.C. (2017). Fatty acid, phospholipid and sterol compositions of breadfruit (*Artocarpus altilis*) and wonderful kola (*Buchholzia coriacea*) seeds. Int. J. Sci..

[25] Yoshida H., Tomiyama Y., Yoshida N., Shibata K., Mizushina Y. (2010). Regiospecific profiles of fatty acids in triacylglycerols and phospholipids from adzuki beans (*Vigna angularis*). Nutrients.

[26] Wang W., Rochfort S. (2024). Comprehensive analysis of phospholipids in pumpkin (*Cucurbita maxima*) seeds: Comparison with cashew nuts and almonds. J. Food Compos. Anal..

[27] Kim K.-S., Lee Y.-H., Jang Y.S., Choi I.-H. (2013). Analysis of fatty acid compositions and biodiesel properties of seeds of woody oil plants in Korea. Korean J. Plant Resour..

[28] Sanchez J., Mangat P.K., Angeles-Shim R.B. (2019). Weathering the cold: Modifying membrane and storage fatty acid composition of seeds to improve cold germination ability in upland cotton (*Gossypium hirsutum* L.). Agronomy.

[29] Duan B., Shin J.A., Lee K.T. (2024). Quantitative analysis of nervonic and erucic acids in human milk: Comparison with infant formula with different fat sources and nutritional stages. J. Oleo Sci..

[30] Kazaz S., Miray R., Lepiniec L., Baud S. (2022). Plant monounsaturated fatty acids: Diversity, biosynthesis, functions and uses. Prog. Lipid Res..

[31] Lee J.M., Lee H., Kang S., Park W.J. (2016). Fatty acid desaturases, polyunsaturated fatty acid regulation, and biotechnological advances. Nutrients.

[32] Tian J., Tian L., Chen M., Chen Y., Wei A. (2022). Low temperature affects fatty acids profiling and key synthesis genes expression patterns in *Zanthoxylum bungeanum* maxim. Int. J. Mol. Sci..

[33] Lee S., Lee Y.B., Kim H.S. (2013). Analysis of functional components of various soybeans. J. Korean Soc. Food Sci. Nutr..

[34] Chanioti S., Katsouli M., Tzia C. (2021). β-Sitosterol as a functional bioactive. A Centum of Valuable Plant Bioactives.

[35] Yuan H., Zhang J., Nageswaran D., Li L. (2015). Carotenoid metabolism and regulation in horticultural crops. Hortic. Res..

[36] Rodriguez-Amaya D.B. (2016). Food Carotenoids: Chemistry, Biology and Technology.

[37] León-López L., Escobar-Zúñiga Y., Milán-Carrillo J., Domínguez-Arispuro D.-M., Gutiérrez-Dorado R., Cuevas-Rodríguez E.-O. (2020). Chemical proximate composition, antinutritional factors content, and antioxidant capacity of anatomical seed fractions of *Moringa oleifera*. Acta Univ..

[38] Kim M.J., Hong C.O., Nam M.H., Lee K.W. (2011). Antioxidant effects and physiological activities of pumpkin (*Cucurbita moschata* Duch.) extract. Korean J. Food Sci. Technol..

[39] Huang D., Ou B., Prior R.L. (2005). The chemistry behind antioxidant capacity assays. J. Agric. Food Chem..

[40] Kedare S.B., Singh R.P. (2011). Genesis and development of DPPH method of antioxidant Assay. J. Food Sci. Technol..

[41] Saini R.K., Prasad P., Sreedhar R.V., Naidu K.A., Shang X., Keum Y.S. (2021). Omega-3 polyunsaturated fatty acids (PUFAs): Emerging plant and microbial sources, oxidative stability, bioavailability, and health benefits—A review. Antioxidants.

[42] Cianciosi D., Forbes-Hernández T.Y., Afrin S., Gasparrini M., Reboredo-Rodríguez P., Manna P.P., Zhang J., Nabavi S.M., Battino M. (2022). The reciprocal interaction between polyphenols and other dietary compounds: Impact on bioavailability, antioxidant capacity and other physico-chemical and nutritional parameters. Food Chem..

[43] Arivarasu L. (2023). In-Vitro antioxidant potential of beta-sitosterol: A preface. Cureus.

