# Supplementary material for: Comparative Profiling of Fat-Soluble Nutrients and Antioxidant Indices in Seeds of Six Maple (Acer) Types
Source: Foods. 2026 Apr 8;15(8):1279. doi: 10.3390/foods15081279 (PMC13114884; doi:10.3390/foods15081279)
Supplement: Supplementary file 1 [file foods-15-01279-s001.zip › foods-4171841-supplementary.pdf]

Supporting Information

Comparative Profiling of Fat-Soluble Nutrients and Antioxidant Indices in Seeds of Six Maple (*Acer*) Types

Sunleng Chhoeun [1,2], Sunyoung Lim [1,2], Jeung-Hee Lee [3] and Jung-Ah Shin [1,2,*]

1 Department of Marine Convergence Science, Kangwon National University, 7 Jukheon-gil, Gangneung 25457 , Gangwon-do , Republic of Korea; chhu-oen.sunleng@kangwon.ac.kr (S.C .); isy7516@kangwon.ac.kr (S.L.)

2 2 Department of Food Processing and Distribution, Kangwon National University, 7 Jukheon-gil, Gangneung 25457, Gangwon-do, Republic of Korea

3 3 Department of Food and Nutrition, Daegu University, Gyeongsan 38453, yeongsangbuk-do Republic of Korea ; jeunghlee@daegu.ac.kr

* Corresponding author.

E-mail addresses: jashin@kangwon.ac.kr; Tel.: +82-33-640-2339

Supplementary Table

Table S1. Taxonomic assignment (as labeled) and seed coat status of the six market-derived *Acer* seed samples.

Sample Number	Sample code	Common name used (manuscript)	Scientific name (as labeled)	Designation (as labeled)	Seed coat status	Country of origin (as labeled)	Seed images
1	RM	Red maple	*Acer palmatum var. amoenum*	-	Seed coat intact	Korea	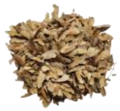
2	M	Maple	*Acer pseudosieboldianum*	(Pax.) Kom.	Seed coat intact	Korea	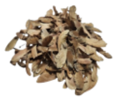
3	GM	Gorosoe	*Acer mono*	Maxim.	Seed coat intact	China (Imported product)	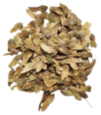
4	TMW1	Three-flowered maple	*Acer triflorum*	Kom.	Seed coat intact	Korea	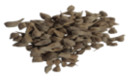
5	TMW2	Three-flowered maple	*Acer triflorum*	Kom.	Seed coat removed (dehulled)	Korea	-
6	JRM	Jeju red maple	*Acer palmatum var. amoenum*	Jeju	Seed coat intact	Korea	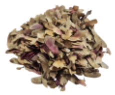

Note: Samples were purchased as commercial products. “With seed coat” indicates intact seeds with the seed coat, whereas “without seed coat” indicates seed coat removal (dehulled). Scientific names and origin information are reported as stated on product labeling. In the case of the three-flowered maple (TMW, *Acer triflorum* Kom.), the seed coat is relatively hard, requiring manual separation of the seed coat and the inner kernel (TMW1 and TMW2). Each component was then homogenized separately using a blender for further analysis. Consequently, TMW1 was analyzed **with** the seed coat intact, while TMW2 was analyzed after the seed coat was removed. In contrast, other maple samples had relatively soft seed coats, allowing for homogenization of the whole seeds without prior separation during the analysis. The taxonomic names and sample labels shown in this table were checked for consistency with the terminology used throughout the main manuscript.

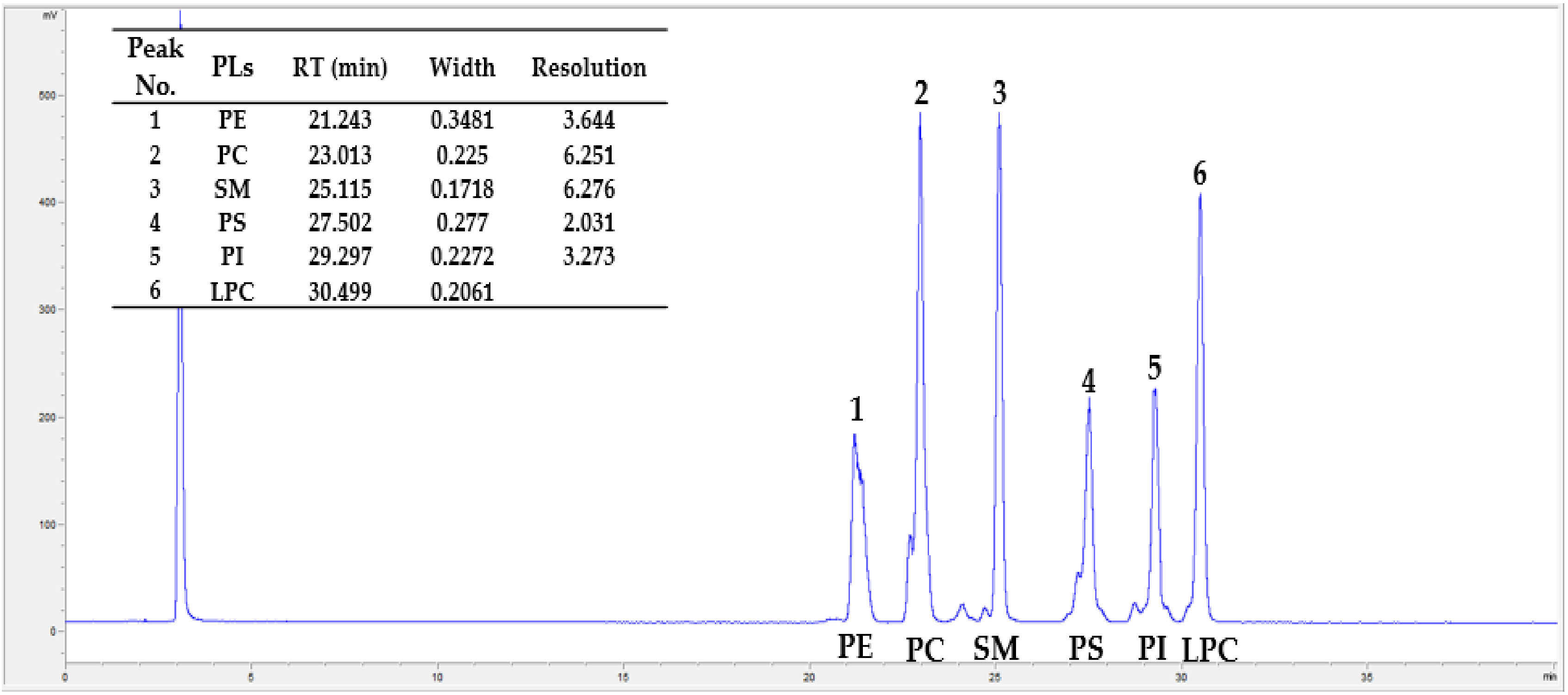

Peak No.	PLs	RT (min)	Width	Resolution
1	PE	21.243	0.3481	3.644
2	PC	23.013	0.225	6.251
3	SM	25.115	0.1718	6.276
4	PS	27.502	0.277	2.031
5	PI	29.297	0.2272	3.273
6	LPC	30.499	0.2061	

Figure S1. Representative HPLC-ELSD chromatogram of phospholipid classes. Peak assignments based on retention times were as follows: 1, phosphatidylethanolamine (PE); 2, phosphatidylcholine (PC); 3, sphingomyelin standard (SM); 4, phosphatidylserine (PS); 5, phosphatidylinositol (PI); 6, lysophosphatidylcholine (LPC).

Note: Resolution (Rs) was calculated as follows: $Rs = 1.18(RT2 - RT1)/(W1 + W2)$
where RT1 and RT2 are the retention times of two adjacent peaks, and W1 and W2 are their baseline peak widths.

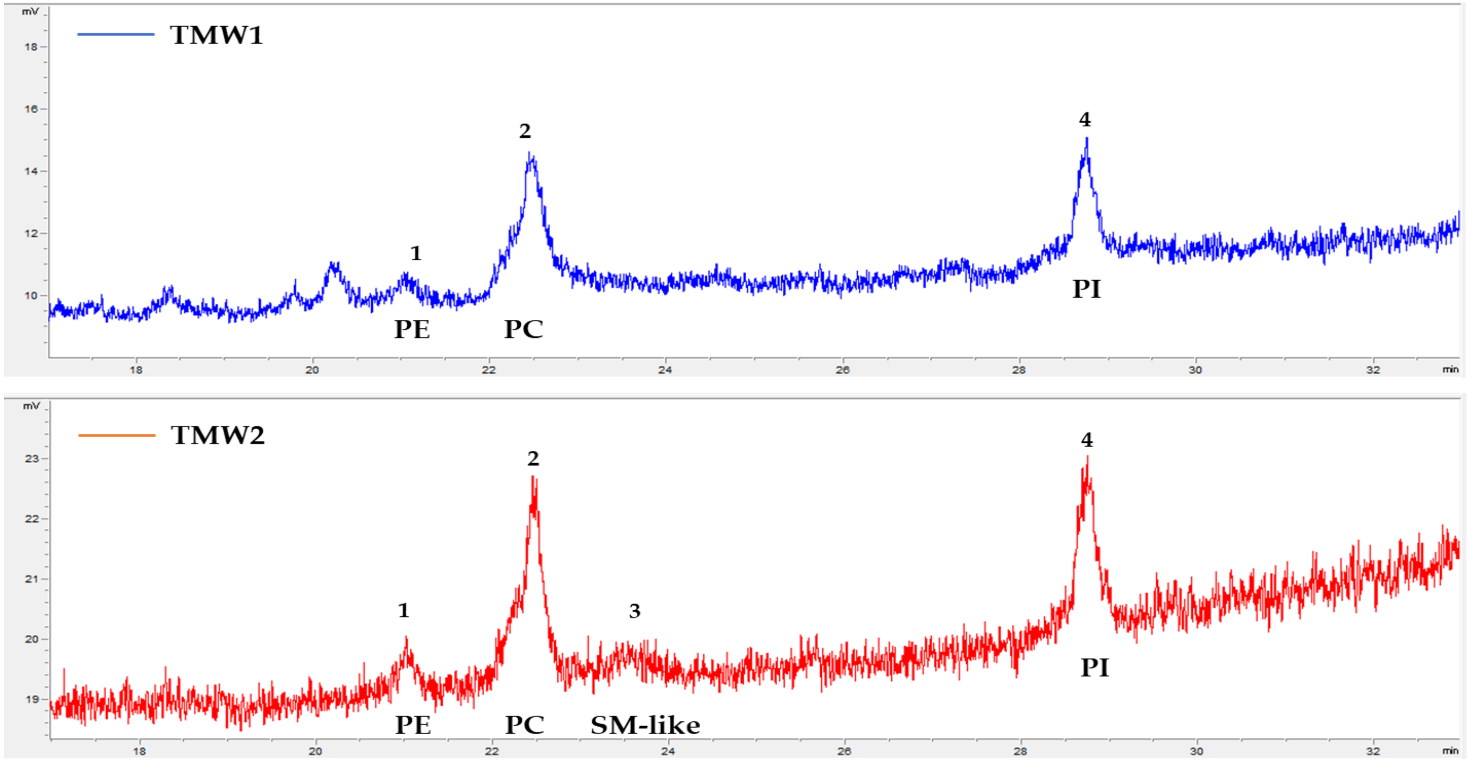

Figure S2. Representative HPLC-ELSD chromatograms of phospholipid classes in TMW1 (*Acer triflorum* Kom.; blue trace, upper chromatogram) and TMW2 (*Acer triflorum* Kom.; red trace, lower chromatogram). Peak assignments were based on standard retention times: 1, phosphatidylethanolamine (PE); 2, phosphatidylcholine (PC); 3, putative SM-like peak; 4, phosphatidylinositol (PI). A peak corresponding to the putative SM-like signal was detected in TMW2, whereas no corresponding peak was detected in TMW1.

Supplementary Figures S1 and S2 provide representative chromatograms and peak-identification materials supporting the phospholipid assignments discussed in the main text.
